# Enzymatically inactive procaspase-1 stabilizes the ASC-pyroptosome

**DOI:** 10.1186/1546-0096-13-S1-O79

**Published:** 2015-09-28

**Authors:** R Stein, MC Heymann, F Kapplusch, S Russ, W Staroske, A Rösen-Wolff, SR Hofmann

**Affiliations:** 1 Department of Pediatrics, Medizinische Fakultät Carl Gustav Carus, Technische Universität Dresden, Dresden, Germany; 2 École Polytechnique Fédérale de Lausanne, Lausanne, Switzerland; 3 Biotechnology Center, Technische Universität Dresden, Dresden, Germany

## Introduction

Caspase-1 (or interleukin-1 converting enzyme, ICE) plays an important role in mediating proinflammatory innate immune responses, especially by activation of pro-IL-1ß within inflammasomes. Some patients with recurrent febrile episodes and systemic inflammation of yet unknown origin harbor *CASP1*-mutations with incomplete penetrance. These *CASP1*-variants cause reduced enzymatic activity of procaspase-1 and less IL-1ß secretion.

## Objectives

The paradox of reduced IL-1β secretion but increased inflammation led to the hypothesis, that *CASP1*-variants have different protein interaction clusters and thus enhance alternative signaling pathways.

## Material and methods

We established an in vitro model of transduced immortalized murine macrophages, expressing either wild type (WT) or enzymatically inactive (C284A) procaspase-1 fusion-reporter proteins and characterized them after NLRP3-inflammasome stimulation.

## Results

As expected, variant procaspase-1 (C284A) macrophages did not secret IL-1ß and pyroptosis was reduced. In addition, the usage of fluorophore-tagged fusion proteins revealed a longer and more intense interaction of the enzymatically inactive procaspase-1 (C284A) with ASC (apoptosis-associated speck-like protein containing a CARD) compared to WT. Variant procaspase-1 (C284A) and ASC formed macromolecular complexes in the cytosol (so called pyroptosomes), that were significantly larger than those formed in cells expressing fluorophore-tagged WT procaspase-1. We could confirm our results by adding the caspase-1 inhibitor YVAD-CMK to Casp1-WT macrophages: the pyroptosomes became larger, more intense and more stable over time. Furthermore, life-cell-imaging detected for the first time, that pyroptosomes of enzymatically inactive procaspase-1 were spread by cell division.

## Conclusion

Variant procaspase-1 stabilizes inflammasome/pyroptosome formation. This may enhance inflammation via two IL-1ß-independent mechanisms: The pyroptosome causes a proinflammatory stimulus through increased recruitment and interaction of further proinflammatory proteins (e.g. RIP2, receptor interacting protein 2). Moreover, this stimulus might be amplified via pyroptosome- and cell division.

